# Evaluating the Mechanical Properties and Energy Absorption Performance of Fluorite Lattice Structures Inspired by Pomelo Peel

**DOI:** 10.3390/ma19142995

**Published:** 2026-07-11

**Authors:** Nghia-Danh Nguyen, Shyh-Chour Huang

**Affiliations:** 1 Faculty of Engineering and Technology, Nguyen Tat Thanh University, 300a Nguyen Tat Thanh, Ward 13, District 4, Ho Chi Minh City 70000, Vietnam; danhnn@ntt.edu.vn; 2 Department of Mechanical Engineering, National Kaohsiung University of Science and Technology, Kaohsiung 807618, Taiwan

**Keywords:** lattice structure, bio-inspired, finite element method, energy absorption, mechanical properties

## Abstract

**Highlights:**

**The main findings are as follows:**

- An appropriate slope and diameter for the struts will help improve the mechanical properties of hybrid lattice structures such as HPFS.
- Improvements in relative density, number of unit cells, and impact speed will improve the performance and stability of the structure’s energy absorption.
- HPFS improves energy absorption by 3.25 times compared to the original FS structure and outperforms other typical lattice structures.

**The implications of the main findings are as follows:**

- They provide reference values for surveying and optimizing geometric parameters affecting the mechanical properties of hybrid lattice structures.
- They provide reference values regarding the influence of factors such as relative density, number of unit cells, and impact speed on energy absorption efficiency.
- They show good potential for application in structures requiring high energy absorption while maintaining mechanical properties, such as the crash box in automobiles.

**Abstract:**

This study proposes a hybrid variant of the fluorite lattice structure inspired by the porous architecture of pomelo peel to enhance mechanical properties and energy absorption performance. The effective mechanical properties of the structure were determined through volume homogenization and optimized using a constrained search and sorting method. The energy absorption behavior under axial crushing was investigated through finite element simulations by varying geometric parameters and comparing the proposed structure with several representative lattice topologies. The results indicate that the elastic modulus increases with increasing strut radius while maintaining a slope parameter λ of 0.6. In addition, higher relative density, a greater number of unit cells, and higher compaction velocity were found to improve the predicted energy absorption capability and deformation stability of the structure. Compared with several conventional lattice configurations, the proposed structure exhibits superior specific energy absorption within the investigated design space. However, the material model neglects strain-rate sensitivity and lacks experimental validation; therefore, the reported results are limited to the adopted numerical framework.

## 1. Introduction

Lattice structures have recently attracted considerable attention due to the excellent mechanical properties and material performance they offer. These structures are typically characterized by their periodic arrangement and linkages via ordered struts, resulting in high isotropy [1]. This demonstrates the great potential of lattice structures in engineering applications that require special capabilities, such as energy absorption [2], sound absorption [3], thermal management [4], vibration absorption [5], or enhancing the flow density of fuel cells [6], while minimizing relative density. Currently, with the development of additive manufacturing (AM) techniques (typically SLA [7] or SLM [8]), modeling complex lattice structures (such as BCC [9], FCC [10] or Octet [11]) has also become simpler and is being applied in many fields, such as aerospace [12], automotive [13], construction [14], medicine [15] or civil engineering [16].

In recent years, traditional strut-shaped lattice structures have gradually been replaced due to certain limitations, such as low absorption efficiency and lack of stability in the plateau phase [17]. Improvements to lattice structures have been made in many directions, such as truss [18], foam [19], hollow [20], hierarchical [21], or hybrid [22]. Liming Zhang et al. [23] proposed a three-dimensional negative Poisson ratio foam metamaterial to improve energy absorption performance for car crashes. The results showed that the structure reduced the maximum crushing force by 25% and improved energy absorption by 23%. Yiajian Ye et al. [24] reinforced the vertical struts for BCC to improve the mechanical properties of the structure. The results showed that the new lattice structures could improve the specific energy absorption by up to 60%. Qiao Zhang et al. [25] proposed a hollow BCC structure with a variable cross-section to improve energy absorption. The results showed that the new structure could improve energy absorption by up to 135.6% compared to the original structure. In general, each development direction has its own advantages and disadvantages; a suitable lattice structure should be found depending on the specific application purpose. However, hybrid lattice structures show good application potential because they combine the advantages and improve the disadvantages of different geometries. In addition, recent works by Giorgio et al. [26] and Fedele et al. [27] show that lattice-like structures can be effectively described by quadratic elastic models, thereby capturing effects such as size-dependent stiffness, non-classical strain propagation, and micro-component interactions, opening up a potential research direction for this topic in the future.

On the other hand, biological structures in nature have continuously evolved and improved over millions of years to adapt to many extremely harsh living conditions. A typical example is the shell of the nautilus, which consists of thin layers of aragonite interspersed with soft protein layers, helping to absorb and disperse energy upon impact [28]. Another example is the bones of animals, whose structure is a combination of the rigidity of hydroxyapatite and the flexibility of collagen, making the structure both strong and capable of withstanding bending and impact [29]. These biological characteristics have inspired many studies to improve metamaterials for multi-field applications in engineering, such as Ngoc Ha San et al. [30], who proposed a hierarchical multi-cell tube inspired by leaf veins. The results showed that the energy absorption capacity of the structure was improved by 55% compared to the conventional structure. Deepak Sharma et al. [31] proposed improving the core of the cylindrical tube with a structure of Euplectella aspergillum sponge to reduce the weight of the medium impact box while maintaining performance. The results showed good application potential of the structure in automotive impact boxes. Hay Miao Zhao et al. [32] proposed improving the hollow BCC network structure with a varied cross-section inspired by bamboo to improve energy absorption performance. The results showed that the elastic modulus of the structure increased by 43.63% compared to the original hollow BCC structure.

**Figure 1:**
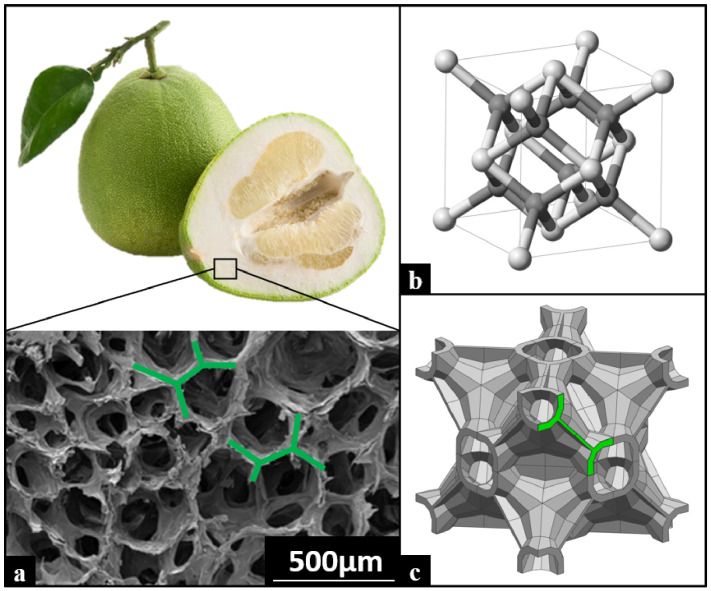
Hollow Pomelo-peel-inspired Fluorite Structure: (**a**) SEM of pomelo peel; (**b**) fluorite structure; (**c**) Hollow Pomelo-peel-inspired Fluorite Structure.

Grapefruits can weigh up to 6 kg and grow on branches up to 15 m high. When dropped by external forces, they can still maintain their structural integrity, showing good energy absorption [33]. This energy absorption is entirely due to the structure of the pomelo peel. This pomelo peel is a collection of interconnected, hierarchically structured, porous, dogbone-like thin sheets, making it a potential material for energy absorption applications (Figure 1a) [34]. Zhi Zhang et al. [35] proposed a multifaceted metamaterial inspired by pomelo peel to enhance energy absorption. The results showed that the structure’s own energy absorption capacity was superior to that of its competitors. Hay Zhang Wen et al. [36] took inspiration from the microstructure of pomelo peel to improve the hierarchical honeycomb structure to enhance the structure’s crush resistance and energy absorption. The results showed that the specific energy absorption capacity and equivalent plateau stress were increased by 1.5 and 2.5 times, respectively, compared to the original honeycomb structure.

Recognizing the potential for good energy absorption through pomelo peel structure, this study proposes a hybrid (combining foam and hollow) variant of the fluorite lattice structure (Figure 1b) inspired by the porous structure of pomelo peel, capable of high energy absorption (Hollow Pomelo-peel-inspired Fluorite Structure—HPFS) (Figure 1c). Based on the volume homogenization method, the study evaluates the mechanical properties of the structure by varying key geometric parameters. Then, using conditional search and constraint methods, the optimal HPFS structure will be evaluated for energy absorption performance by examining the geometric parameters characteristic of the crystal lattice and comparing it with other lattice structures to assess its potential. This study introduces a new metamaterial with high mechanical and energy absorption properties for practical engineering applications such as automotive crash boxes.

## 2. Design and Material

### 2.1. Pomelo-Peel-Inspired Fluorite Structure Generation

**Figure 2:**
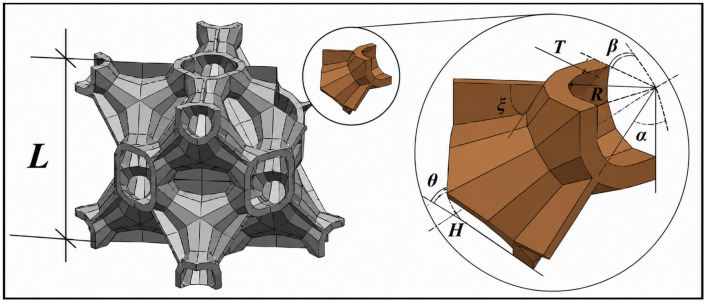
Geometry parameters of HPFS.

The fluorite lattice structure is a variant of the FCC structure, consisting of struts linked together at the vertices and the centroid of a regular tetrahedron. The original FS structure has high flexibility in its deformation mechanism, and its mechanical properties will be improved if the struts are hollowed out and their thickness is reinforced [37]. However, compared to the ideal mechanical characteristic curve (HS upper bound curve), the performance is not yet optimized [38]. The preliminary shape of the structure is presented in Figure 2. Inspired by the porous structure of pomelo peel, in addition to hollowing out the struts to optimize the relative density of the structure, the study will reinforce them with thin plates to connect the struts more tightly. To avoid reducing the flexibility of the structure, the thickness of these connecting plates will be deliberately controlled. In addition, preliminary research shows that changing the cross-section of the struts from circular to decagonal shape will improve the energy absorption performance of the structure. Furthermore, lattice structures often have high stress concentrations at the nodes, reducing their performance. Therefore, the study proposes creating slopes for the struts, which will help disperse stress concentrations and increase the structural strength.

To aid in later calculations, the study proposes a mathematical equation to determine the volume V of the HPFS structure. Because network structures are often highly symmetrical, it is readily apparent that the HPFS is also formed by 32 small struts. Therefore, the volume V of the HPFS is calculated by subtracting the apical excess volume $V_{2}$ and the radial excess volume $V_{3}$ of the decagonal cylinder $V_{1}$ and adding the additional volume of the connecting wing $V_{4}$.

(1) $$V=32\xi^{*}\left[V_{1}-3\left(V_{2}-V_{3}-V_{4}\right)\right]$$

(2) $$V_{1}=\pi H\left[R^{2}-{\left(R-T\right)}^{2}\right]$$

(3) $$V_{2}=\frac{5}{8}\left(\tan\alpha +\tan\beta \right)\left[R^{3}-{\left(R-T\right)}^{3}\right]$$

(4) $$V_{3}=\frac{\pi }{19}\left(\tan\beta +\tan\theta \right)\left[R^{3}-{\left(R-T\right)}^{3}\right]$$

(5) $$V_{4}=\frac{T\tan\beta }{6}{\left(\frac{L\sqrt{2}}{4}-\frac{R}{\cos\alpha }\right)}^{2}$$

**Figure 3:**
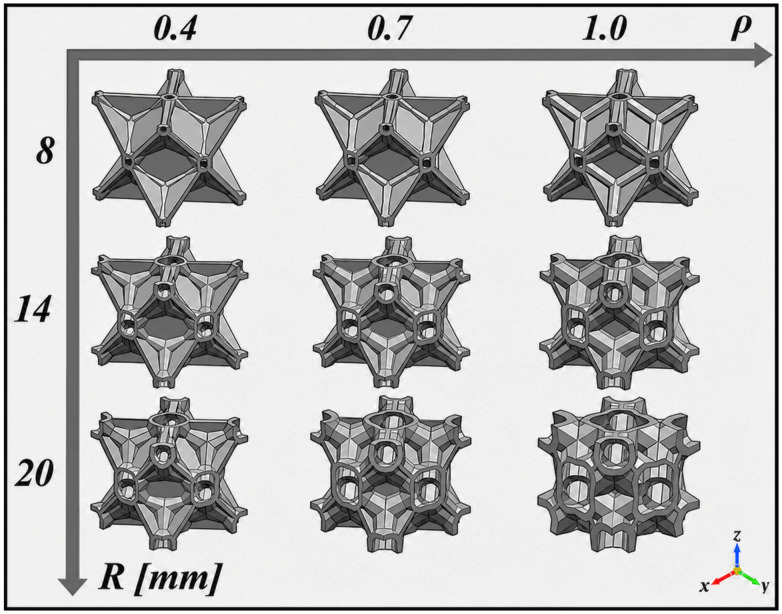
HPFS with different geometric parameter pairs at ρ equal to 0.2.

In this model, L is the unit cell edge dimension, H is the strut height, R is the strut radius, T is the thin-wall thickness, $\xi^{*}$ is the volume addition factor when the slope λ changes, and α/β/θ are the surface inclination angles that determine the volume fractions $V_{2}$ and $V_{3}$ to be subtracted from the cylinder $V_{1}$. The study will evaluate the mechanical properties of HPFS at a relative density ρ of 0.2. R is investigated in the range of 8 mm to 20 mm with a step of 0.25, λ is investigated in the range of 0.3 to 1.0 with a step of 0.05, and T will be controlled to maintain the specified relative density. A total of 375 different HPFS structures will be designed by customizing R, λ, and T (Figure 3).

### 2.2. Homogenization Method

Based on the linear relationship between stress and strain according to Hooke’s law, the effective elastic stiffness tensor $C_{ij}$ of the structure is determined as in Equation (6). For the case of axial compression load, the characteristic elastic constants of the material including the effective elastic modulus (E) and the Zener isotropic coefficient (A) are calculated according to Equation (7) and Equation (8) respectively:

(6) $$C_{ij}=\left[\begin{array}{cccccc} C_{11} & C_{12} & C_{13} & 0 & 0 & 0\\ C_{12} & C_{22} & C_{23} & 0 & 0 & 0\\ C_{13} & C_{23} & C_{33} & 0 & 0 & 0\\ 0 & 0 & 0 & C_{44} & 0 & 0\\ 0 & 0 & 0 & 0 & C_{55} & 0\\ 0 & 0 & 0 & 0 & 0 & C_{66} \end{array}\right]$$

(7) $$E=\frac{\left(C_{11}-C_{12}\right)\left(C_{11}+2C_{12}\right)}{\left(C_{11}+C_{12}\right)}$$

(8) $$A=\frac{2C_{44}}{C_{11}-C_{12}}$$

To determine the optimal mechanical properties of a lattice structure, a constrained optimization problem is established, where the goal is to maximize the elastic modulus (E) while ensuring near-isotropy by constraining the Zener coefficient (A) to approach 1. Specifically, the problem is stated as follows:

(9) $$\begin{array}{c} find\;R,\lambda \\ \min-E=-\frac{\left(C_{11}-C_{12}\right)\left(C_{11}+2C_{12}\right)}{\left(C_{11}+C_{12}\right)}\\ s.t\left\{\begin{array}{c} \left|A-1\right|=\left|\frac{2C_{44}}{C_{11}-C_{12}}-1\right|\leq 10^{-3}\\ V^{*}\leq V_{cons}\\ R_{\min}\leq R\leq R_{\max}\\ \lambda_{\min}\leq \lambda \leq \lambda_{\max} \end{array}\right. \end{array}$$

**Figure 4:**
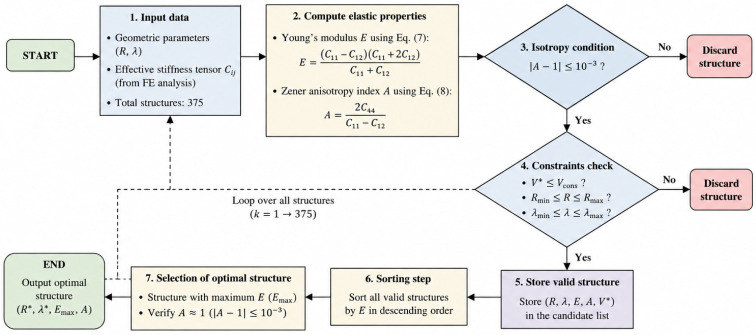
Flowchart of the algorithm for optimizing the mechanical properties of HPFS.

In this case, $V^{*}$ is the relative density of the structure, while R and λ are the geometric parameters controlling the radius and slope of the lattice element, respectively. To solve the above optimization problem, a discrete search algorithm combined with a sorting method was developed and implemented; the computational procedure is presented in Figure 4. In this, the input dataset includes 375 lattice structures, each characterized by a set of geometric parameters (R, λ) and the corresponding effective stiffness tensor $C_{IJ}$, obtained from finite element simulation. For each structure, the characteristic mechanical quantities, including the effective elastic modulus E and the Zener isotropic coefficient A, are calculated based on the stiffness tensor $C_{IJ}$ according to the expressions in Equations (7) and (8). Next, a constraint filtering step is applied to eliminate structures that do not satisfy the near-isotropic condition, specifically A ≈ 1, while ensuring compliance with geometric and relative density constraints. Structures that satisfy all conditions are included in the valid solution set and sorted in descending order of elastic modulus E. Finally, the optimal structure is determined as the structure with the largest E value in this solution set, while still satisfying all the given constraints.

### 2.3. Finite Element Method and Crashworthiness Criterion

**Figure 5:**
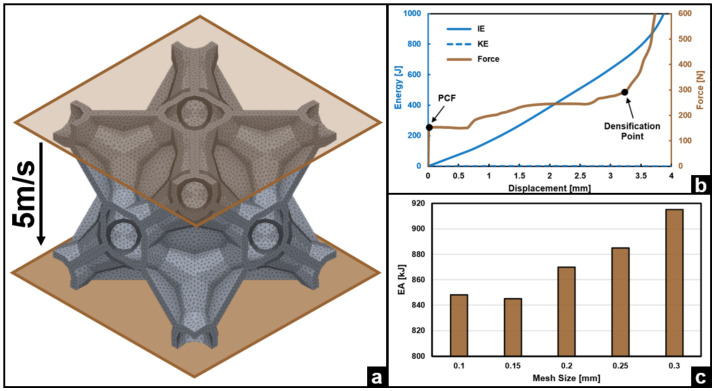
Semi-static compression simulation model setup: (**a**) simulation setup under uniaxial compression load; (**b**) analysis of the speed sensitivity of AlSi10Mg; (**c**) investigation of the convergence of energy absorption capacity of HPFS with different mesh sizes.

After determining the optimal HPFS structure, a numerical study was performed to evaluate the energy absorption characteristics under axial compression loading in a semi-static environment. Finite element simulation was implemented in ABAQUS/Explicit software. The model consists of two rigid plates arranged at the two ends of the structure, where the lower plate is completely fixed, while the upper plate is controlled to move vertically to perform the compression process (Figure 5a). The boundary conditions are set as follows: all degrees of freedom of the lower plate are locked, while the upper plate is only allowed to move in the compression direction. The compression process is controlled by displacement with a travel equivalent to 80% of the initial height of the structure. Contact between surfaces is modeled using the general contact algorithm with a friction coefficient of 0.2, to reflect the actual friction interaction between elements during large deformation [39]. The AlSi10Mg material was modeled using an isotropic elastic–plastic model, with the mechanical parameters presented in Table 1 [40]. Because this study focuses on the effect of compression rate on the geometric deformation mechanism rather than the speed-dependent behavior of the material, the material model does not include the strain rate sensitivity-dependent effect. This decision is supported by the kinetic energy and internal energy (KE/IE) evaluation results, in which the ratio of kinetic energy is always less than 5% of the total energy throughout the simulation (Figure 5b). This indicates that the load state is close to a semi-static condition, and the effect of strain rate on the material response can be ignored without distorting the overall deformation trend. Regarding the failure mechanism, this study does not apply the explicit failure criterion or element deletion. Instead, the degradation of the load-bearing capacity of the structure is described through large plastic deformation, local buckling, and contact interaction between lattice bars. This approach allows for the accurate reproduction of typical geometric collapse mechanisms of lattice structures, such as inclined shear slip and bar bending, which are consistent with experimental observations. The element mesh was constructed using three-dimensional C3D10M bulk elements, suitable for large strain and complex contact problems [41]. Mesh convergence analysis was performed with different element sizes, and a mesh size of 0.15 mm was chosen to ensure a balance between accuracy and computational cost (Figure 5c).

The energy absorption performance of the structure is evaluated through two main indicators, Specific Energy Absorption (SEA) and Energy Absorption Stability Factor (EASF), determined according to Equations (10) and (11). SEA reflects the energy absorption capacity per unit mass, while EASF characterizes the degree of stability of the energy absorption process through the oscillations in the compressive force in the plateau phase [42]. These settings ensure that the numerical model not only accurately reflects the mechanical behavior of the structure but also has high reproducibility, meeting the requirements of transparency and reliability of the simulation method.

(10) $$SEA=\frac{\int_{0}^{d_{\max}}F(x)dx}{m}$$

(11) $$EASF=\frac{PCF}{\int_{0}^{d_{\max}}F(x)dx}d_{\max}$$

In this formula, m is the mass of the lattice structure, PCF is the first maximum load threshold when subjected to axial crushing load, and $d_{max}$ is the energy absorption distance.

### 2.4. Evaluating the Energy Absorption Performance of HPFS

**Figure 6:**
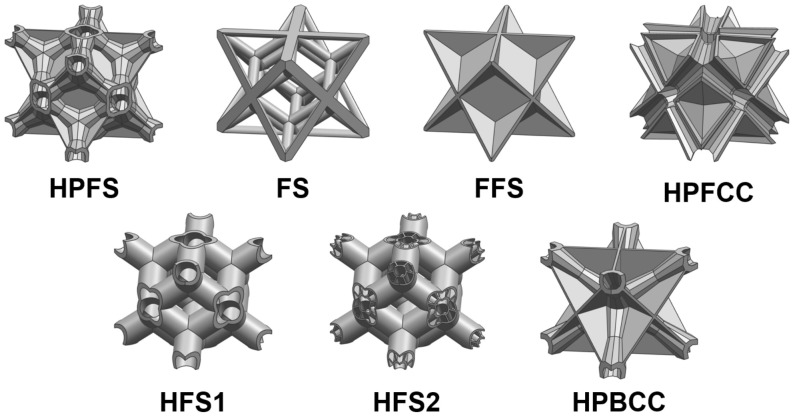
Variations of FS and the application of connecting wings to typical lattice structures.

To systematically evaluate the influence of geometric parameters on the energy absorption performance of HPFS structures, this study investigated three main factors: unit cell size, loading rate, and relative density. Each factor was considered at three different values, as shown in Table 2. This approach allows for quantitative analysis of the sensitivity of mechanical properties to each parameter, while also assessing the interaction between them. In addition, besides evaluating HPFS structures individually, the study also compared them with other typical lattice structures to clarify the relative advantage in energy absorption capacity. Specifically, the selected structures included: FS, FFS, HFS1, HFS2, HPBCC, and HPFCC (Figure 6). These structures represent different deformation mechanisms, from bending-dominated (FS, BCC) to stretch-dominated (FFS, FCC), thereby providing a comprehensive comparative platform for energy absorption performance. To ensure fairness in comparison, all structures were designed to satisfy the same relative density level under each case study. Detailed geometric parameters of each structure, including outer diameter, inner diameter, and wall thickness, are presented in Table 3. Through this approach, the study not only identified the optimal set of geometric parameters for the HPFS structure but also clarified the competitive position of the proposed structure compared to typical lattice topologies under the same operating conditions.

## 3. Results and Discussions

### 3.1. Experimental Validation via Benchmark Studies

**Figure 7:**
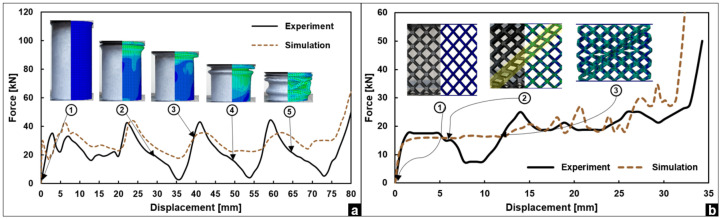
Comparison of experimental and simulation results of force–displacement characteristics of: (**a**) TF; (**b**) BCC.

In this study, the fabrication of prototypes and the performance of actual compression tests on the proposed structure were limited by equipment conditions and costs. Therefore, to ensure the reliability of the finite element model, an indirect validation method through published experimental studies was applied. Specifically, two studies were selected, including the study by Ahmed S. Mohamed et al. [42] on axial compression tests of Tube with a filter (TF) fabricated from AlSi10Mg material, and the study by Wanqi Ma et al. [40] on compression tests of BCC lattice structures also using the same material. These studies provided complete experimental data, including force–displacement curves and detailed descriptions of the deformation mechanism. Based on this information, the corresponding finite element models were reconstructed in ABAQUS with boundary conditions, loads, and material parameters set similarly to those in the original experiments. The simulation results obtained show good agreement with experimental data, both in terms of force–displacement characteristic curve values and overall strain trend. In particular, collapse mechanisms such as local buckling in thin tubes (Figure 7a) and shear-dominated collapse in BCC structures (Figure 7b) are accurately reproduced in the simulation. The similarity between simulation and experimental results in these standard problems shows that the finite element model used in the study is capable of reliably predicting the mechanical behavior of AlSi10Mg lattice structures under compressive load. Therefore, although no direct experiment was performed on HPFS, this benchmark validation method still ensures the reliability and reasonableness of the simulation results [45].

### 3.2. Optimizing HPFS

**Figure 8:**
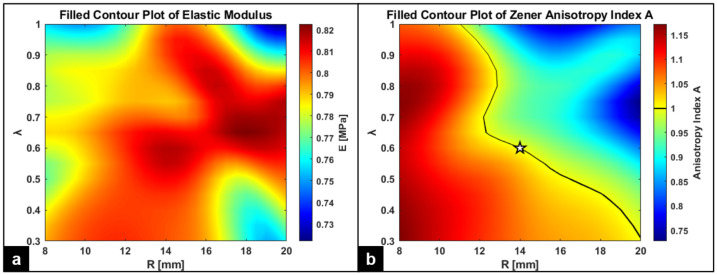
The graphs show the mechanical properties of the HPFS structure when the geometric parameters are changed: (**a**) elastic modulus graph of HPFS; (**b**) Zener anisotropy index graph of HPFS.

This study evaluated the mechanical properties of 375 HPFS samples at a relative density of 0.2. Figure 8a shows the elastic modulus E of HPFS within the surveyed range. The results show that determining the appropriate geometric parameters greatly affects the elastic modulus of HPFS. Specifically, increasing the strut radius R while maintaining the slope λ within the range of 0.5 to 0.8 improves the performance E of HPFS. However, determining an inappropriate slope λ will reduce the performance. Figure 8b shows the A curve of HPFS. The black curve represents A equal to 1, which is the ideal value for lattice structures with good isotropy. The results show that determining the appropriate geometric parameters for HPFS helps improve the isotropy of the lattice structure. In this study, improving the strut radius R must occur simultaneously with a linear reduction in the slope λ to maintain the HPFS in an ideal isotropic condition. To determine the HPFS with optimal mechanical properties, the study used a constrained search and sorting method to determine the optimal E and A, with the constraint that E is maximized and A approaches 1. The results showed that the optimal HPFS has a radius R of 14 mm and a slope λ of 0.6 (star in the figure).

### 3.3. The Influence of Geometric Parameters on the Energy Absorption Performance of HPFS

**Figure 9:**
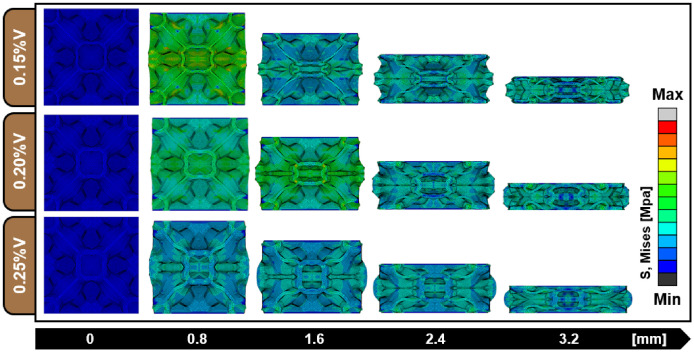
Deformation mechanism of HPFS with improved volumetric density under axial crushing load.

After determining the optimal geometric parameters affecting the mechanical properties of HPFS, the study continued to evaluate the energy absorption performance of HPFS by improving other geometric parameters characterizing the lattice structure, such as relative density, number of unit cells, and impact velocity. Figure 9 illustrates the geometric deformation mechanism of HPFS through improvements in relative density. The results show that at a relative density of 0.15, HPFS has a high stress concentration in the central region and spreads horizontally, causing this region to be the first to be misaligned. As compression continues and the folds at the center saturate, the stress gradually spreads to the foam sheets, reinforcing the struts and causing shape misalignment. This deformation process continues, with stress concentration distributed relatively evenly across the structure, before transitioning to the densification state. Improving the relative density changes the deformation tendency of HPFS. In this design, stress is concentrated in the central area and spreads outwards in an arc shape along the longitudinal direction. This makes the central area less prone to deformation, thus enhancing its load-bearing capacity during a collision.

**Figure 10:**
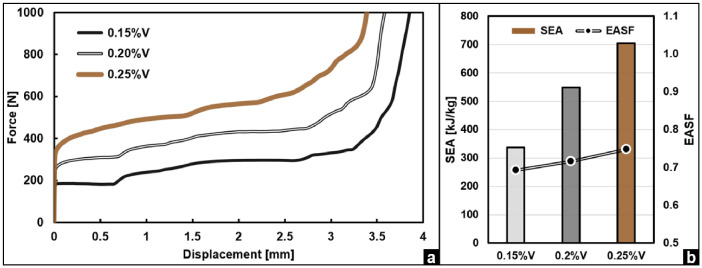
Evaluating the energy absorption performance of HPFS through relative density improvement: (**a**) force–displacement characteristic curve of HPFS with relative density improvement; (**b**) SEA and EASF of HPFS with relative density improvement.

**Figure 11:**
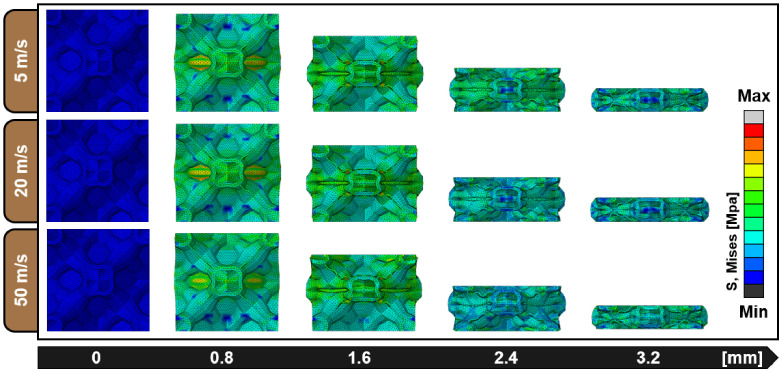
The deformation mechanism of HPFS when the loading rate is increased.

Figure 10a shows the force–displacement characteristic curve of HPFS with improved relative density. The results show that at a relative density of 0.15, the PCF of HPFS is relatively low, not increasing sharply or decreasing abruptly compared to traditional impact-resistant structures, but maintaining this value at a stable level of approximately 200 N. The later stage of the plateau state increases slightly linearly compared to the initial stage of the pre-densification state. Increasing the relative density will enhance the load-bearing capacity of HPFS. However, the energy absorption state at the plateau stage will be slightly reduced because the densification stage occurs earlier. Figure 10b evaluates the SEA and EASF of HPFS with improved relative density. The results show that improving the relative density will enhance the SEA and EASF, which tend to stabilize at 0.75. Figure 11 illustrates the deformation regime of HPFS as the loading rate increases. The results show that below 20 m/s, there is not much change in the deformation mechanism of the structure. The deformation mechanism of HPFS tends to be uniform and symmetrical; HPFS maintains an early folding state at the center and spreads horizontally because stress is concentrated in this central region. When the loading rate is increased to 50 m/s, the deformation mechanism loses its symmetry in the top–bottom region (the region directly and indirectly subjected to impact). Stress is concentrated more in the upper region of the HPFS, causing the deformation process to occur earlier than in the lower region. As the deformation process in the upper region gradually saturates, deformation begins to occur in the lower region before the structure completely transitions to the densification state.

**Figure 12:**
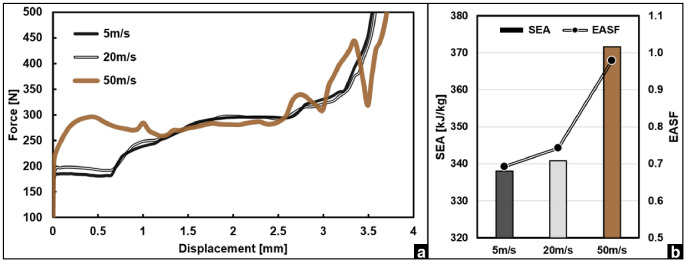
Evaluating the energy absorption performance of HPFS by increasing the loading rate: (**a**) force–displacement characteristic curve of HPFS as the loading rate increases; (**b**) SEA and EASF of HPFS as the loading rate increases.

Figure 12a shows the force–displacement characteristic curve of HPFS under different loading rate. The results show that when the loading rate is below 20 m/s, the force-displacement characteristic curve does not differ significantly. This indicates relatively good stability of the structure under loading rate outside of semi-static conditions. When the loading rate reaches 50 m/s, the load-bearing capacity of the structure in the initial stage of the absorption process is significantly enhanced. The force–displacement characteristic curve tends to be horizontal, although some instability still exists during the impact. Figure 12b shows the SEA and EASF of HPFS as the loading rate increases. The results show that the higher the loading rate of HPFS, the greater its specific energy absorption capacity. At the same time, EASF gradually becomes ideal as the stability during energy absorption approaches level 1.

**Figure 13:**
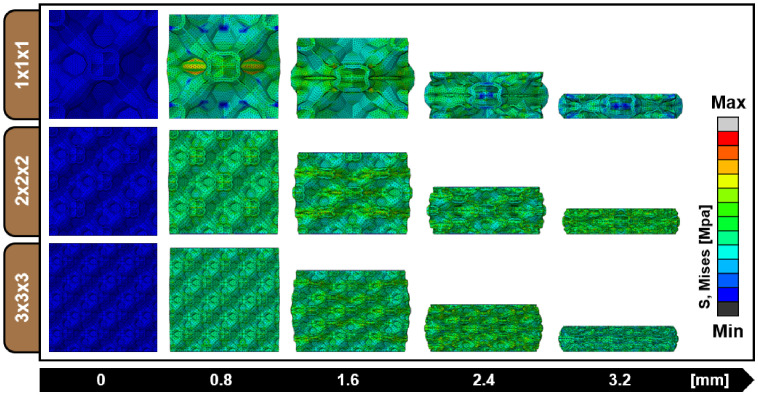
The deformation mechanism of HPFS when the number of unit cells increases.

Figure 13 illustrates the strain regime of HPFS as the number of unit cells increases. The results show that increasing the number of unit cells helps to distribute concentrated stress more effectively. Because the structure is highly isotropic, the strain regime at the unit cells also becomes similar. In this process, shape distortion occurs early at the center of the intersections and spreads horizontally to the surrounding area. When the folds in this area are saturated, the reinforcing foam sheets continue to bear the load and perform folding before the concentrated stress disperses throughout the area and transitions to densification.

**Figure 14:**
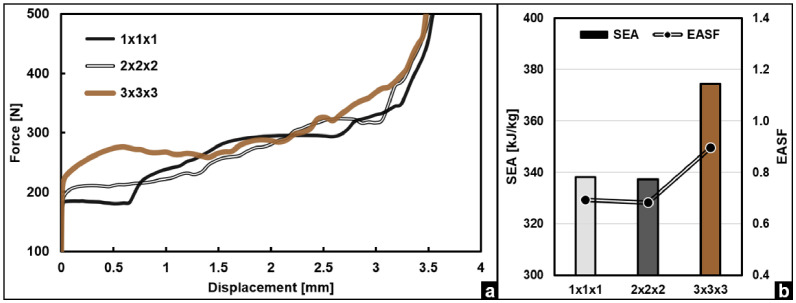
Evaluating the energy absorption performance of HPFS by increasing the number of unit cells: (**a**) force–displacement characteristic curve of HPFS with an increasing number of unit cells; (**b**) SEA and EASF of HPFS with an increasing number of unit cells.

Figure 14a shows the force–displacement characteristic curve of HPFS as the number of unit cells increases. The results show that the load-bearing capacity of HPFS tends to increase linearly and more stably when the structure has 2 × 2 × 2 unit cells. When the number of unit cells is increased to 3 × 3 × 3, the load-bearing capacity of HPFS improves at the initial impact stage. However, the maintenance of load-bearing capacity at 280N does not last until the end of the impact; instead, it begins to increase linearly at a compression stroke of 2 mm. This reduces the stability of HPFS during energy absorption. Figure 14b shows the SEA and EASF of HPFS as the number of unit cells increases. The results show that increasing the number of unit cells improves the specific energy absorption capacity and the stability of the structure during energy absorption.

### 3.4. Evaluating the Potential of HPFS

#### 3.4.1. When Compared to Other Variants of FS

**Figure 15:**
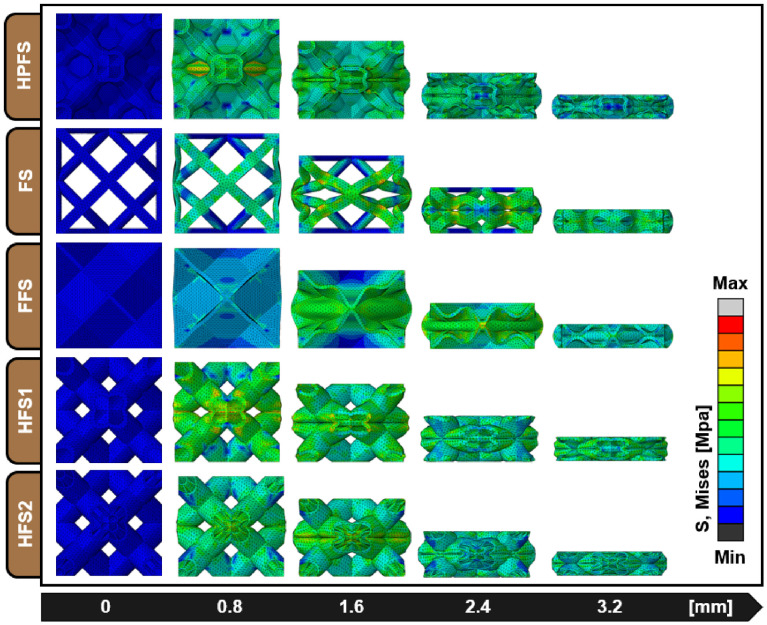
Deformation regime of fluorite lattice structure variants.

To assess the potential of HPFS for energy absorption structures, this study compared HPFS (a hybrid foam and hollow structure) with other variations of fluorite lattice structures, including traditional strut structures, foam, hollow, and horsetail-inspired structures. Figure 15 illustrates the deformation regime of the different fluorite lattice structure variations. It can be seen that the traditional strut structure exhibits uneven stress distribution, with large differences between regions, and stress concentrated at the intersections of the struts, causing deformation in these areas. The foam structure shows better stress distribution in the early stages of impact. However, deformation is strong, and stress is concentrated in the central region in the later stages of impact. Uneven stress distribution may lead to reduced energy absorption efficiency. Foam and horsetail-inspired structures show similarities in their shape and deformation mechanism. Stress is concentrated at the intersection of the struts in the central area, causing the initial flexing before dispersing to the upper and lower areas of the structure. HPFS shows that combining the shapes (foam and hollow) improves the deformation regime of the structure. At the initial stage of impact, stress is concentrated in the central area, where the foam sheets have good load-bearing capacity, while other areas have more evenly distributed stress compared to other FS variants. In the later stage of impact, HPFS overcomes the excessive stress concentration in the central area that causes rapid shape collapse compared to foam structures. This indicates that HPFS has relatively better uniformity and stability compared to other FS variants.

**Figure 16:**
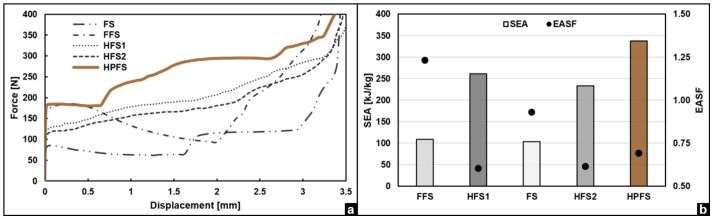
Comparison of energy absorption performance of different fluorite lattice structure variants: (**a**) force–displacement characteristic curves of fluorite lattice structure variants; (**b**) SEA and EASF of HPFS and other variants of fluorite lattice structure.

Figure 16a shows the force–displacement characteristic curves of the FS variants. The force–displacement characteristic curve of FS-Truss is relatively stable, but its load-bearing capacity is not good. FS-Foam has relatively good load-bearing capacity in the initial impact phase. However, its load-bearing capacity collapses rapidly afterward, and it soon enters a densification state. FS-Hollow shows slightly better load-bearing capacity than FS-Horsetail, although the stress concentration of FS-Horsetail is more evenly distributed. This suggests that increasing the number of thin walls of the structure instead of the thickness of the thin walls sometimes reduces the load-bearing capacity of the structure. HPFS shows relatively good load-bearing capacity, with the development of the force–displacement characteristic curve showing a combination of both foam and hollow forms. In this case, the structure exhibits a similar load-bearing capacity to FS-Foam at the initial impact stage. However, its load-bearing capacity does not degrade; instead, it increases linearly, similar to FS-Foam. Figure 16b compares the SEA and EASF of fluorite variants. Although the energy absorption stability of FS-Truss is relatively good, its specific energy absorption efficiency is not high. While FS-Foam has relatively good initial load-bearing capacity, its lack of stability results in poor specific energy absorption efficiency. FS-Hollow is slightly better in terms of energy absorption efficiency than FS-Horsetail. HPFS shows superior specific energy absorption efficiency compared to other structures. Specifically, the SEA of HPFS is 338 kJ/kg, 3.25 times higher than that of the traditional strut-like structure. Nevertheless, the stability during energy absorption is somewhat lower. To overcome this problem and improve the stability of HPFS during energy absorption, the study proposes a strategy of slightly reducing the mechanical properties of the structure (reducing E and keeping A = 1). However, this may slightly reduce the energy absorption efficiency of HPFS.

#### 3.4.2. When Compared to Other Lattice Structures

**Figure 17:**
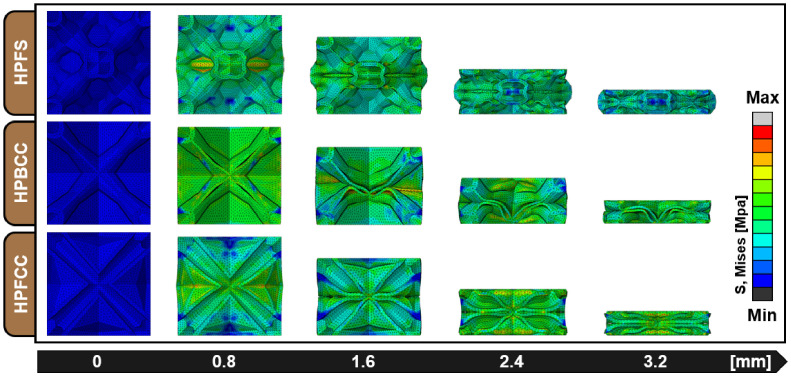
The deformation regime of typical lattice structures inspired by pomelo peel.

The study further evaluated the energy absorption potential of HPFS compared to other lattice structures, BCC and FCC, under axial crushing load. Figure 17 shows the strain regime of the pomelo-peel-inspired lattice structures. It can be seen that HPFS exhibits a more uniform and stable strain regime compared to its edge-peel counterparts. In the early stages of compression, HPBCC has relatively better elemental stress concentration than the other structures. However, in the later stages of compression, uneven shape misalignment may affect the energy absorption performance of the structure compared to the others. Although the strain regime of HPFCC is relatively uniform, the uneven stress distribution may affect the energy absorption performance of the structure.

**Figure 18:**
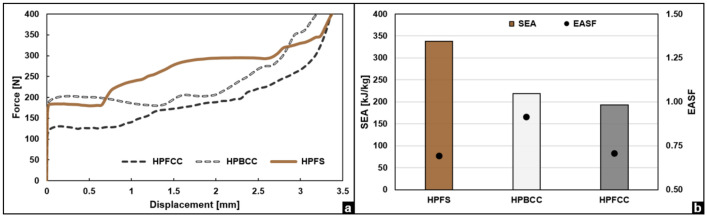
Comparison of energy absorption performance of typical lattice structures treated with pomelo-lo-peel-inspired technology: (**a**) force–displacement characteristic curves of lattice structures treated with pomelo-lo-peel-inspired technology; (**b**) SEA and EASF of HPFS and lattice structures treated with pomelo-peel-inspired technology.

Figure 18a compares the force–displacement characteristics of pomelo-peel-inspired lattice structures. The results show that the initial load-carrying capacity of HPBCC is relatively good and stable. However, the densification state of HPBCC begins earlier than that of other structures. HPFS has a slightly lower load-carrying capacity than HPBCC at the initial stage of impact. However, at a compression stroke of 0.8 mm, the load-carrying capacity of HPFS begins to increase linearly compared to HPBCC. Although HPFCC has a relatively stable force–displacement characteristic, its load-carrying capacity is somewhat inferior to its competitors. Figure 18b compares the SEA and EASF of pomelo-peel-inspired lattice structures. The results show that the SEA of HPFS is approximately 1.5 times higher than that of other lattice structures. Nevertheless, the energy absorption stability of HPFS is somewhat lower than that of HPBCC.

#### 3.4.3. Comparing the Mechanical Properties of HPFS with Other Structures

**Figure 19:**
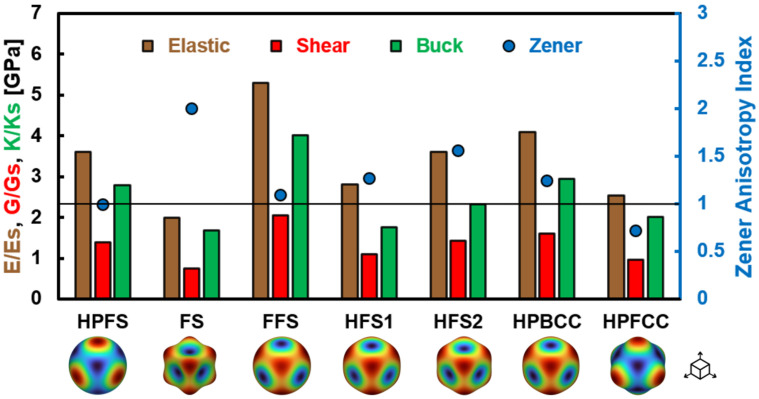
Comparison of the mechanical properties of HPFS with typical crystal structures at a relative density of 0.2.

To clarify the mechanical advantages of the proposed structure, the mechanical properties of HPFS were compared with other typical structures, including elastic modulus (E), shear modulus (G), buck modulus (K), and Zener anisotropy index (A). The results are shown in Figure 19. The Zener index (A) of the HPFS structure is approximately 1, indicating the near-isotropic nature of the effective material. Compared to the other structures, which exhibit significant deviations from the isotropic state (A ≠ 1), HPFS is capable of uniformly distributing stress in the spatial directions. This is particularly important in energy absorption problems, where the applied load is often not entirely in a single direction. Furthermore, the E, G, and K values of HPFS are in the intermediate range compared to the structures investigated. Specifically, HPFS does not reach the maximum value like stretch-dominated structures (e.g., FFS or HPBCC), but it is also not as low as typical bending-dominated structures (e.g., FS). This balance offers a significant advantage: the structure is stiff enough to maintain its initial load-bearing capacity, while remaining sufficiently “soft” to allow for stable deformation progression, thereby improving energy absorption efficiency and preventing sudden collapse. Furthermore, the elastic modulus distribution of HPFS shows a relatively uniform geometry with less variation compared to other structures. This demonstrates that the load-bearing capacity of HPFS is not strongly dependent on the direction of the load, in contrast to highly anisotropic structures where the elastic modulus varies significantly in direction. This characteristic contributes to the improved mechanical stability of the structure under complex loading conditions. In summary, the above results show that the HPFS structure achieves an optimal balance between isotropy and effective stiffness. As a result, the structure not only ensures reliable load-bearing capacity but also facilitates the evolution of deformation, a key factor in improving energy absorption efficiency compared to traditional lattice structures.

## 4. Conclusions

This study proposes a hybrid variant of the fluorite lattice structure inspired by the porous structure of pomelo peel. Using volume homogenization, the mechanical properties of HPFS were optimized by controlling geometric parameters. Furthermore, under axial crushing load, the energy absorption performance of HPFS was evaluated by improving geometric parameters and comparing it with other potential lattice structures. Some conclusions are as follows:

- The mechanical properties of HPFS are optimized with a strut radius of 14 mm and a slope λ of 0.6.
- Increasing the number of unit cells, relative density, and impact speed improves energy absorption performance and stability.
- HPFS improves SEA by 3.25 times compared to traditional structures. However, energy absorption stability is reduced.
- HPFS exhibits superior energy absorption performance compared to other typical lattice structures.

The results highlight the potential of the proposed bio-inspired hybrid fluorite structure for energy absorption applications, such as protective components in transportation and impact-mitigation systems. Nevertheless, several limitations should be acknowledged. The constitutive material model used in this study does not incorporate strain-rate sensitivity, and the numerical results were not directly validated against experimental crushing tests. Consequently, the observed influence of compaction velocity and the optimal design parameters should be interpreted within the scope of the adopted modeling assumptions. Future work will focus on incorporating rate-dependent material behavior and conducting experimental validation to further assess the applicability of the proposed structure under realistic loading conditions.

## Figures and Tables

**Table 1:** Properties of ALSi10Mg material.

Factor	Unit	Value
Density	kg/m^3^	2670
Young’s modulus	MPa	70.0
Poisson’s ratio	-	0.33
Yield strength	MPa	230.0
Ultimate Strength	MPa	370.0
Elongation at break	%	15

**Table 2:** Factors and levels of use for evaluating the energy absorption performance of HPFS.

Factors	Level 1	Level 2	Level 3
Unit cell	1 × 1 × 1	2 × 2 × 2	3 × 3 × 3
Loading rate [m/s]	5	20	50
Relative density	0.15	0.20	0.25

**Table 3:** Geometric parameters of typical lattice structures.

Lattice Structures	Outer Diameter [mm]	Inner Diameter [mm]	Thickness [mm]	Relative Density [%V]
HPFS	1.4	1.192	0.0693	0.2
FS [43]	0.692	-	-	
HFS1 [32]	1.25	0.967	0.189	
HFS2 [37]	1.25	0.625	0.086	
FFS [44]	-	-	2.485	
HPBCC	1.4	1.1605	0.05987	
HPFCC	1.4	1.2365	0.08175	

## Data Availability

The original contributions presented in this study are included in the article. Further inquiries can be directed to the corresponding author.
